# PROTOCOL: Group‐based interventions for posttraumatic stress disorder: A systematic review and meta‐analysis of the role of trauma type

**DOI:** 10.1002/cl2.1328

**Published:** 2023-05-13

**Authors:** Siobhán M. Griffin, Alžběta Lebedová, Elayne Ahern, Grace McMahon, Daragh Bradshaw, Orla T. Muldoon

**Affiliations:** ^1^ Centre for Social Issues Research, Department of Psychology University of Limerick Limerick Ireland

## Abstract

This is the protocol for a Campbell systematic review. The primary objective is to assess the effects of group‐based treatments on posttraumatic stress disorder (PTSD) symptomology in people diagnosed with PTSD (by a clinician or screening instrument) or referred to a PTSD treatment group for their symptoms by a medical professional. We will also examine a range of moderators that may affect the efficacy of group‐based treatments, including the nature of the trauma (interpersonal, stigmatized) and the group fit (in terms of gender and shared vs. unshared trauma). Further, we will also explore what, if any, group‐based and social identity factors are recorded and how they relate to PTSD outcomes.

## 1 BACKGROUND

### 1.1 The problem, condition or issue

Incidence of posttraumatic stress disorder (PTSD) varies across populations, countries, and trauma types (Kessler et al., 2017; Liu et al., 2017), with rough estimates suggesting about 3.5% of the population experience PTSD at some point in their lifetime (Kessler et al., 2005). Often PTSD is a chronic and life debilitating condition (Hidalgo & Davidson, 2000). Posttraumatic stress disorder involves four clusters of symptoms relating to: re‐experiencing symptoms of a distressing event (e.g., intrusive thoughts), hyperarousal (e.g., irritability, increased startle response), avoidance of any reminders of the distressing event, and numbing and/or negative moods or cognitions associated with the event, as defined by the fifth edition of the *Diagnostic and Statistical Manual of Mental Disorders* (DSM‐5; American Psychiatric Association, 2013). Symptoms of PTSD or posttraumatic stress have been reported after a variety of traumatic and distressing events including, but not limited to, sexual violence (Amone‐P'olak et al., 2018), childhood abuse (Barlow et al., 2017), military combat and deployment (Armenta et al., 2019; Campbell et al., 2019), the COVID‐19 pandemic (Tyra et al., 2021; Xiong et al., 2020), motor vehicle accidents (Blanchard et al., 1995), exposure to violence (Cuartas & Roy, 2019; Trevillion et al., 2012), mass shootings (Bardeen et al., 2013), and natural disasters (An et al., 2019; Muldoon et al., 2017).

The experience of PTSD symptoms has far reaching consequences. People who experience PTSD often experience co‐morbid psychiatric conditions (Pietrzak et al., 2011), report a lower quality of life and poorer functional outcomes (Magruder et al., 2004; Schnurr et al., 2006; Zatzick et al., 2002), and are at increased risk for suicide (Guerra & Calhoun, 2011; Sareen et al., 2007). Furthermore, PTSD incurs a high economic burden, both in terms of direct and indirect costs (Dams et al., 2020; McGowan, 2019; von der Warth et al., 2020).

Effective means to treat PTSD symptoms remains an important challenge; treating PTSD optimally can help improve well‐being and reduce the economic burden on society by reducing associated indirect costs associated with impaired functioning at work and in social domains (e.g., absenteeism). However, PTSD treatment guidelines differ in their consensus of what treatments are efficacious, particularly the utility of group treatments for treating PTSD. For instance, some guidelines do not address group interventions (American Psychological Association, 2017; National Institute for Health and Care Excellence NICE, 2018), while other guidelines provide recommendations for certain types of group therapy. As an example, the International Society for Traumatic Stress Studies guidelines (International Society for Traumatic Stress Studies [ISTSS], 2018) state that trauma‐focused group cognitive behaviour therapy (CBT) is effective but there is insufficient evidence to make recommendations for other types of group treatments (e.g., group interpersonal therapy). Indeed, a meta‐analysis by Lewis and colleagues (2020), which informed the current ISTSS recommendations, showed that group‐based treatments with a trauma focus reduced PTSD symptoms (compared to waitlist controls and/or care as usual conditions) but evidence was mixed for group therapies that were not trauma focused (e.g., group relaxation, psychoeducation, psychodynamic therapy). Although the Department of Veterans Affairs and Department of Defense (VA/DoD) (2017) provides a moderate recommendation for group therapy, these guidelines do not specify what type of group therapy. Further, attention to the group processes that might foster therapeutic effects is lacking.

It appears from these guidelines that CBT‐based group therapies are an effective way of treating PTSD symptoms, but it is unclear what other types of group treatments for PTSD are of value, for who, and what group processes are involved. It is also worth noting that these recommendations primarily stem from the results of a handful of randomized controlled trials (RCTs; Hamblen et al., 2019). A key aspect, overlooked by research to date, is attention to the role of shared group membership and the associated group‐based social identity processes that groups can offer; a limitation that this review and meta‐analysis will address.

The social identity approach to health demonstrates that shared group memberships (e.g., belonging to a support group, same gender identity group, group membership shaped by a shared type of trauma) can affect psychological health, both positively (Cruwys, Haslam, et al., 2014) and negatively (Kellezi & Reicher, 2012). Shared group memberships can offer people psychological resources through processes such as social identification with others in the group, group cohesion, perceived fit with others, and/or a sense of common fate (Borek et al., 2019; Steffens et al., 2021). The psychological resources offered through groups (e.g., belonging, social support, felt understanding) may help buffer the effects of trauma and alleviate PTSD symptoms. However, at times shared group membership might serve as a social curse (e.g., if it is a stigmatized identity) and this can worsen psychological outcomes, such as PTSD, as it can undermine belonging with the group (e.g., Muldoon, Walsh, et al., 2019). These processes remained underexplored as potential mechanisms through which group therapies may have implications for PTSD treatment. The proposed review will therefore synthesize existing evidence, including both RCTs and non‐RCTs, and will investigate factors that may contribute to the efficacy of group‐based treatments.

### 1.2 The intervention

Group‐based interventions can offer an effective way to reduce PTSD symptoms (J. G. Beck & Coffey, 2005; Sloan et al., 2013). Existing interventions for PTSD, such as CBT (J. G. Beck et al., 2009), dialectical behaviour therapy (Bradley & Follingstad, 2003), psychotherapy (Classen et al., 2011), and cognitive processing therapy (Chard, 2005) are often conducted in group format and can effectively reduce PTSD symptoms. Although the type of treatment taking place within these groups is often the sole focus of the researchers' investigations, evidence has shown that the provision of psychological treatments for PTSD in a group format can alleviate PTSD symptoms to a greater degree compared to waitlist controls and/or care as usual conditions (for meta‐analyses see; Barrera et al., 2013; Schwartze et al., 2019; Sloan et al., 2013). While individual and group‐based therapies may include similar content and psychological approaches, groups offer further potential by harnessing the benefits of the social group. Furthermore, economically, provision of psychological treatments in a group format are more cost and time effective than individual‐level treatment (from a clinician standpoint) as multiple people can be treated at once by either one or two facilitators who lead the sessions (e.g., J. G. Beck & Coffey, 2005).

### 1.3 How the intervention might work

Regardless of the specific therapeutic approach used in a group treatment, we argue that it is the *group‐based* nature of the treatment that is most beneficial, and this may offer advantages over individual‐level treatments. This hypothesis stems from the social identity approach to health which emphasizes the importance of group memberships for everyday life (Tajfel & Turner, 1979; Turner et al., 1987). Being a member of a group is thought to be beneficial for psychological well‐being and health (Cruwys et al., 2014; C. Haslam et al., 2008; Jetten et al., 2014) as groups can provide individuals with social, psychological, and material resources to cope with the adverse effects of life change, including trauma (C. Haslam et al., 2018; Jones et al., 2012; Kearns et al., 2018; Muldoon et al., 2019; Walsh et al., 2015)

For instance, belonging to a social group has been shown to be associated with lower risk of depression (Cruwys et al., 2013; Sani et al., 2012; Seymour‐Smith et al., 2017), greater well‐being (Iyer et al., 2009; Sani et al., 2015), and lower PTSD symptoms (Jones et al., 2012; Muldoon & Downes, 2007). As such, group‐based psychological treatments for PTSD may offer additional benefits for well‐being through the provision of psychological resources, such as a sense of belonging and social support (Avanzi et al., 2018; C. Haslam et al., 2016; S. A. Haslam et al., 2005; Walter et al., 2016).

The provision of social support is particularly important for people who have experienced trauma, as often the experience of trauma leads to social withdrawal (Hofmann et al., 2003), and sometimes results in the loss of valued group memberships and identity loss (Muldoon et al., 2019). Further, trauma can influence a person's ability to engage with and bond with others (Charuvastra & Cloitre, 2008), serving to amplify the isolation experienced. This is particularly evident in traumas that are stigmatized in society (Kellezi & Reicher, 2012). Social isolation can in turn affect the severity of PTSD symptoms as a lack of social support can impair a person's ability to regulate their distress, leading to poorer clinical outcomes (Price et al., 2018). Group‐based treatments may help provide valuable social support for individuals who may be facing isolation as a result of their trauma, and ultimately improve clinical outcomes.

Moreover, group‐based treatments can put people in contact with others ‘like them’ (i.e., others who have also experienced a similar trauma). Being part of a group may foster social identification with the group (i.e., the degree to which a person feels the group positively informs their definition of self; Postmes et al., 2013). Greater social identification with the group provides resources such as acceptance, perceived efficacy, support, and solidarity (Cruwys & Gunaseelan, 2016), in an effect known as the social cure (Jetten et al., 2012). Importantly, identification with others in the group is believed to help mitigate the effects of trauma and reduce PTSD symptoms (Muldoon et al., 2019).

However, it is worth noting that group‐based treatments might not always be beneficial. Some researchers have raised concerns that hearing about another person's trauma might be retraumatising for some, or that disclosing traumatic experiences in a group setting might lead to unhelpful social comparisons, such as feeling that one's trauma is not as legitimate as other group members (J. G. Beck & Coffey, 2005; Courtois & Ford, 2012). Similarly, reliving or confronting traumatic memories in a group format might be uncomfortable for some people (Røberg et al., 2018). This has implications for the efficacy of group‐based treatments as confronting traumatic memories is thought to be a key process in treating PTSD symptoms. In this regard, it may be the case that individual‐based therapies might show advantages over group‐based therapies.

### 1.4 Why it is important to do this review

Research to date evaluating the efficacy of group‐based interventions to treat PTSD has presented mixed evidence. Some research demonstrates that group‐based interventions effectively lower PTSD symptoms in comparison to control conditions (e.g., waitlist control, treatment‐as‐usual; Barrera et al., 2013; Schwartze et al., 2019; Sloan et al., 2013). However, compared to individual‐level treatments, group‐based interventions have been shown to either be less effective (e.g., Ehring et al., 2014) or have similar effects on PTSD symptoms (e.g., Bisson et al., 2013; Schwartze et al., 2019; Sloan et al., 2013). Indeed, as previously highlighted, current policies and guidelines differ in the degree to which they recommend group‐based treatment formats (e.g., International Society for Traumatic Stress Studies ISTSS, 2018; Phoenix Australia Centre for Posttraumatic Mental Health, 2020). A consideration of the role of shared group membership and social identity processes that groups can harness may elucidate the efficacy of group‐based interventions for treating PTSD. It may be the case that the effectiveness of group‐based interventions depends on a number of considerations, specifically: (i) group‐based factors (e.g., reported social support from group members, identification with other group members, facilitator characteristics), (ii) whether all group members have experienced a similar trauma (or a range of different traumas), (iii) if the trauma has stigma or shame attached, (iv) if the reason for the trauma was the result of intentional human action, and (v) the gender‐composition of the group (mixed, all female, all male) in traumas with a clear gender dimension (e.g., sexual assault).

First, an up‐to‐date synthesis is needed comparing the efficacy of group‐based psychological interventions to waitlist/usual care conditions, and to comparable individual‐level treatment (i.e., the same psychological treatment approach is used), that includes both RCTs and non‐RCTs.

Second, a number of group processes and factors are argued to bring about positive changes in well‐being. For instance, a meta‐analysis found that individuals who reported greater social identification with their therapy group demonstrated greater well‐being (Steffens et al., 2021). Likewise, research on group treatments targeting anxiety, depression, and eating disorder symptoms have shown that individuals who reported greater identification with their group showed better outcomes (Cruwys et al., 2014; McNamara & Parsons, 2016). Borek et al. (2019) identified a number of group‐based factors associated with success in health‐behaviour‐change interventions which may have important implications for group‐based PTSD treatments, including group cohesion, group climate, group engagement, social support, and facilitator characteristics (e.g., warmth, relatedness, demographics). These studies highlight a number of important considerations that influence outcomes. Therefore, in this review we will describe any group‐based and social identity factors reported on within each study and how they relate to PTSD symptomology. This may include, but is not limited to, perceived social support from other group members, measures of identification with other group members, likability of the group facilitator, and similarity between the group participants and the facilitator.

Third, the *fit* between the person and the other group members is an important factor in driving identification with the group (Cruwys et al., 2020), and thus the ability to benefit from the psychological and material resources groups offer (Jetten et al., 2012). In groups where individuals have all experienced a similar trauma this fit may (or may not) be better. Further, when all members of a group have shared a similar experience (‘a common fate’) this may foster greater social identification through a range of mechanisms including shared experience, shared values, solidarity, self‐categorization, shared fate, and a sense of ‘in‐group’ membership (Acharya & Muldoon, 2017; Drury, 2012; Muldoon et al., 2019). It may be the case that the effects of group treatments for PTSD vary between groups that are comprised of people who have all experienced the same trauma, and groups where individuals have experienced a range of different traumas. A consideration of the group composition in this way may be revealing in terms of treatment outcomes and therefore will be explored in this review.

Fourth, research has shown that experiencing a trauma with stigma attached to it can undermine psychological well‐being further. Importantly, while diversity exists in cultural definitions of stigma, there is also remarkable consistency. Across many cultures and countries, certain experiences are universally associated with stigma. These include issues pertaining to sex and sexuality, infectious diseases/illnesses, and unfamiliar people and practices. A large body of research outlines the processes through which stigma can cause harm to health. Corrigan et al. (2011) proposed the most widely accepted model explaining the damage caused by stigma—the progressive model of self‐stigma. This model distinguishes between enacted stigma and felt stigma. It is the latter that has the most powerfully negative consequences. Those affected by a stigmatized trauma are often aware of the stereotypes relating to their trauma ambient in their culture and then may also agree with it, apply it to themselves, and suffer harm as a result. In many regards then, this conceptualization of stigma is strongly related to stereotype endorsement of affected groups themselves—they themselves see their stigma as it were. For the purposes of this review, we conceptualize stigmatizing traumas in this regard, encompassing traumas such as rape, intimate partner violence, bereavement by suicide, and childhood sexual abuse. This is in comparison to other traumas which are less commonly associated with stigma (e.g., physical assault that is not domestic violence, witnessing an assault, bereavement due to accidents or illness, motor vehicle accidents, falls, natural disasters).

An examination of the efficacy of group‐based treatments for stigmatizing traumas (vs. less stigmatizing traumas) is warranted considering conflicting evidence on group membership and stigma. On one hand, if all members of a group have experienced the same stigmatized trauma this can actually motivate people to engage with the group, which can have subsequent positive effects on well‐being (Bradshaw & Muldoon, 2020; Lichtenstein, 2003). However, other research has shown that sometimes individuals may negatively self‐stigmatize themselves or actively resist identifying with a stigmatized group, undermining any sense of belonging or connection with the group (Jetten et al., 2013; Muldoon et al., 2019; Quinn & Earnshaw, 2013). This then can negatively impact on well‐being and increase distress, an effect termed the ‘social curse’ (Kellezi & Reicher, 2012). In this review, where groups are comprised of members who have all suffered a similar trauma, we will explore if stigma plays a role in treatment outcomes.

Fifth, past research demonstrates that traumas originating from intentional human acts, such as rape, combat exposure, childhood neglect, childhood physical abuse, sexual molestation, and physical assault, are associated with the highest rates of PTSD (Alisic et al., 2014; American Psychiatric Association, 2013; Charuvastra & Cloitre, 2008; Cloitre et al., 2019; Frans et al., 2005; Kessler et al., 1995). It is thought that these traumas in particular can compromise a person's ability to engage and bond with others, and undermine their sense of trust in others (Charuvastra & Cloitre, 2008). This may have implications for the effectiveness of group‐based interventions in treating PTSD symptoms, as many group treatments involve participants sharing their thoughts and emotions with others in the group setting which typically requires some degree of trust. As such, research is needed to examine if trauma *type* (caused by intentional human actions vs. not caused by intentional human actions) moderates group treatment outcomes.

Sixth, the gender composition of treatment groups must be considered for traumas that have a gender dimension, for instance in the case of rape or domestic violence. From a social identity perspective, it may be beneficial for group members to be of the same sex so that members share potentially important and relevant social identities (S. A. Haslam et al., 2012; Muldoon et al., 2019). Indeed, two previous meta‐analyses have indicated that the gender composition of the group is an important consideration. The results demonstrated that group‐based interventions were more effective in alleviating PTSD symptoms in *all female* groups (Schwartze et al., 2019; Sloan et al., 2013). Notably, in most of these studies (with all female groups), the trauma experienced was gendered in nature (e.g., involved unwanted sexual contact, physical assault, sexual assault, childhood sexual abuse). This finding warrants further exploration to examine whether the gender composition of the treatment group affects clinical outcomes when the trauma is gendered.

Seventh, and finally, past research has suggested that the optimal group size for group treatments is six to eight people (Yalom, 1995). However, little research tests this recommendation. In the present meta‐analysis, we will examine group size as a potential moderator.

The results of this systematic review and meta‐analysis will inform policy and practice in the treatment of PTSD by providing insights into which conditions within group‐based therapies for PTSD may be beneficial or detrimental for treatment outcomes. We build on the work of other recent systematic reviews which focused on group‐based psychotherapeutic formal change theory interventions led by trained group leaders (Schwartze et al., 2019) and RCT treatments for PTSD (Lewis et al., 2020). We augment this prior work by including: any studies with a group‐based intervention for PTSD regardless of the therapeutic approach taken, studies that are clinician‐led and peer‐led, and studies which do not use a RCT‐design. The proposed review will provide an up‐to‐date synthesis of existing evidence and extend previous research by examining a range of moderators that may affect the efficacy of group‐based treatments, including the nature of the trauma (interpersonal, stigmatized) and the group fit (in terms of gender and shared vs. unshared trauma). We also explore what, if any, group‐based and social identity factors are recorded and how they relate to PTSD outcomes. As this review includes comparisons between group‐based treatments, individual treatments, and/or no treatment, we will explore if comparison type (individual vs. no treatment) has an effect. The results will facilitate policy makers in coming to informed decisions surrounding treatment recommendations.

## 2 OBJECTIVES

The primary objective is to assess the effects of group‐based treatments on PTSD symptomology in people diagnosed with PTSD (by a clinician or screening instrument) or referred to a PTSD treatment group for their symptoms by a medical professional. The objectives targeted by this review are:

1. To compare the efficacy of group‐based interventions compared to waitlist/usual care and/or minimal attention conditions that take place on an individual basis (i.e., not group‐based).
2. To compare the efficacy of group‐based interventions compared to comparable individual‐based treatment.
3. To assess what social identity or group‐based factors (if any) are measured and whether they are associated with the outcomes of interest.
4. Where possible, assess whether the efficacy of group‐based interventions is moderated by: shared experience of similar trauma (whether all members of the group experience the same trauma or different traumas), stigma (if the trauma/s experienced have societal stigma attached), the nature of the trauma (if the trauma was caused by the intentional actions of another person/s or not), gender composition of the group (mixed gender grouping, all female, all male), comparison type (individual‐based treatment or no treatment), and group size (<6, 6‐8, >8).
5. To assess if there are any negative implications/adverse outcomes related to group‐based treatments (e.g., worsening of PTSD symptoms, occurrence or worsening of other psychiatric symptoms such as depression and suicidal ideation, and/or increased or new alcohol or substance use).

## 3 METHODS

### 3.1 Criteria for considering studies for this review

#### 3.1.1 Types of interventions

##### Group‐based intervention

Group‐based treatments can include a variety of treatments including group CBT, group counselling, group psychodynamic therapy, and other group treatment formats. Consistent with a review by Schwartze et al. (2019), group‐based treatment protocols must include a minimum of three individuals per group and must plan to meet for at least five sessions to be included. Interventions must assess PTSD symptomology pre‐intervention (before or at the start of the first session) and post‐intervention (either at the last session or after a follow‐up period) using a valid and reliable measure. Subsequent follow‐up measurements will also be included in the review, but more than one post‐intervention measurement is not required for consideration in the review. Interventions can be delivered by a clinician or can be peer‐led. Participants must not be receiving any other psychological treatments for PTSD (e.g., individual therapy outside of the study). However, studies that include participants taking medication for PTSD (or related distress) are eligible for inclusion. To be considered for sub‐group analyses, the type of trauma experienced by participants must be explicitly stated in the study (or provided by the researchers when contacted for this information). Studies without this information may be included to examine the overall effects of treatment type on PTSD outcomes but will not be subject to subgroup analyses by trauma.

Sometimes, in the delivery of group treatments, participants may attend individual‐based sessions with a clinician. If so, we will note this and note how many individual sessions these participants received. If many studies include group‐based treatments that also have individual sessions, sensitivity analyses will be conducted to see if this influenced any observed results. While we will examine, where available, if group processes (e.g., social connectedness, group cohesion) influence treatment outcomes, it is not a requirement for a study to report on these indices.

##### Control or comparison condition

Comparison conditions can either include (i) a comparable active psychological individual‐based treatment, and/or (ii) a control condition. In this regard, comparison individual‐based treatments can include, but are not limited to, individual CBT, cognitive processing therapy, eye movement sensitization, stress inoculation training, individual counselling, psychodynamic therapy, and other individual treatments provided that this type of psychological treatment is comparable to the treatment employed in the group. In other words, both the individual and group conditions need to employ a similar treatment (i.e., both use CBT, both use psychodynamic therapy). If dissimilar treatments are used in the group condition and individual treatment condition (e.g., group CBT vs. individual psychodynamic therapy) these conditions will not be compared in the meta‐analysis. Control conditions can include, but are not limited to, waitlist groups, treatment‐as‐usual/usual care/standard care, symptom monitoring, and minimal attention control groups; the control condition must take place on an individual‐level basis (i.e., not a group‐based format). If any control condition occurs in a group format, we will include this study and narratively describe the difference between this and the group treatment, but this study will not be included in the analyses (i.e., group treatment vs. individual treatment, group treatment vs. individual control). Group‐based control conditions are not comparable to individual‐based control conditions as we believe groups themselves offer benefits. If at least two studies employ group‐based control groups, then we will examine this as a separate comparator (i.e., group treatment vs. group‐based control). It may also be the case that a study includes two or more group‐based interventions. These will be considered in analyses if an appropriate comparison group (as specified) is also included within the study design.

#### 3.1.2 Types of studies

All objectives will focus on quantitative evidence only. Participants must be assigned to either a group‐based treatment or a comparison condition. The comparison condition can take a number of forms including an active comparable individual‐based psychological treatment and/or a control condition. We anticipate that the number of RCTs matching the inclusion criteria may be limited. Therefore, non‐RCT studies that include a comparison condition will be considered, for instance: (i) quasi‐RCTs where a quasi‐random method of allocation is employed (e.g., the order of recruitment), (ii) studies with a matching design to establish condition equivalence, or (iii) participants are not randomly assigned but baseline equivalence is ensured (e.g., via matching, statistical controls, or equivalence on PTSD symptoms), if not the study needs to provide results from which baseline‐adjusted effect sizes can be calculated otherwise the study will be excluded due to a critical risk of bias (see Section 3.3.6 for our definition of critical confounders). Our analyses will therefore be conducted on all studies included, but sensitivity analyses will also be conducted including only RCTS. Studies available in English will be included and may be published or unpublished. Protocols, trial registrations, systematic reviews and meta‐analyses, book chapters, letters to the editor, and conference proceeding will not be included.

#### 3.1.3 Types of participants

Participants must be over 18 years of age at the time of treatment. At least 70% of participants in the sample must have been diagnosed with PTSD, or screened positively for PTSD on a psychometrically valid measure, or have been referred for treatment by a medical professional for their PTSD symptoms (similar to; Bisson et al., 2013; Lewis et al., 2020; Schwartze et al., 2019). No other restrictions in terms of age, location, ethnicity, severity of symptoms, duration of symptoms, length of time since trauma, or geographical location apply. Samples where participants have comorbid disorders will be included, provided that the intervention is primarily aimed towards reducing PTSD symptoms (similar to; Simon et al., 2021).

#### 3.1.4 Types of outcome measures

##### Primary outcomes

The primary outcome is PTSD symptomology or indices of posttraumatic stress symptoms (or clinical/base change), either indexed by self‐reported symptoms using a psychometrically valid questionnaire or diagnosed by a qualified clinician (or both). Only studies with a continuous measure of PTSD symptoms will be included. Examples of validated measures include, but are not limited to, the PTSD Checklist (Blanchard et al., 1996), the International Trauma Questionnaire (Cloitre et al., 2018), and the Impact of Events Scale‐Revised (Weiss & Marmar, 1997). Clinician administered measures will take precedence over self‐reported measures, provided that the data is continuous in nature. Clinician rated measures, such as the Clinician Administered PTSD Scale for DSM‐5 (CAPS‐5; Weathers et al., 2018), are considered gold standard, and preference for this type of measurement is consistent with past reviews (e.g., Bisson et al., 2013; Lewis et al., 2020). Primary outcomes will be narratively described in the full review, with additional quantitative meta‐analyses performed alongside subgroup analyses where appropriate.

##### Secondary outcomes

Secondary outcomes are not required for inclusion in the review, but data will be extracted on secondary outcomes of interest when reported. These include, where relevant, continuous measures of depression, somatic symptoms, and posttraumatic growth (or clinical/base change in these outcomes), as well as any adverse outcomes reported. We will also report descriptively on attrition (drop‐out rates) and loss of PTSD diagnosis (i.e., remission). Validated peer‐reviewed measures of these outcomes will be eligible. For instance, depressive symptoms are often assessed via the Beck Depression Inventory (A. T. Beck, 1961; A. T. Beck et al., 1996), the Patient Health Questionnaire (Kroenke et al., 2001), the Centre for Epidemiologic Studies Depression Scale (Radloff, 1977), the Hamilton Depression Rating Scale (Hamilton, 1960), and the depression subscale of the Hospital and Anxiety Depression Scale (Zigmond & Snaith, 1983). Posttraumatic growth is often assessed using the posttraumatic growth inventory (Cann et al., 2010; Tedeschi & Calhoun, 1996). These outcomes will be extracted for descriptive purposes. However, if a sufficient amount of studies report on depression and posttraumatic growth as outcomes we will run meta‐analyses on these outcomes. We will also consider attrition as an outcome for subgroup analyses if a sufficient number of studies exist to allow for this to be examined. We will then estimate how depression, attrition, and posttraumatic growth are impacted by the group‐based factors previously outlined, specifically: trauma group type (same vs. mixed), stigmatized trauma (or not), if the trauma was caused by intentional human action/s (or not), gender grouping (mixed, all female, all male), and group size.

We will also explore, where possible, how reported group‐based processes such as belonging, group cohesion, and trust, influence the primary outcome (PTSD symptoms). These will be discussed narratively.

#### 3.1.5 Duration of follow‐up

All studies must report on the primary outcome (PTSD symptoms) post‐intervention. This follow‐up can be on the last day of treatment or soon afterwards (within a week/7 days). Where relevant, subsequent follow‐up periods will be examined. There is no limitation on the duration of this follow‐up. However, if large differences exist between study follow‐up times, we will categorize these studies into subgroups for analysis: posttest (immediately after the intervention up to 1 week), short‐term follow‐up (1–12 weeks post‐intervention), medium‐term follow‐up (3–12 months post‐intervention), and longer‐term follow‐up (>12 months). This will provide information on the temporal effects of group interventions on PTSD symptoms.

#### 3.1.6 Types of settings

As we are primarily interested in the role of groups, group‐based interventions that take place in a variety of settings will be included. This is not limited to community‐based settings, hospital settings, in‐patient treatment settings, out‐patient treatment settings, residential treatment settings, community settings, prison settings, and clinical or medical settings. There are no restrictions on country or locality (e.g., rural or urban).

### 3.2 Search methods for identification of studies

To identify studies, electronic databases will be searched and the reference lists of all included studies will be screened for further potentially relevant research. For electronic searches, filters and limiters such as ‘English’, and ‘adults 18+’ will be used where possible, for instance in Ovid Medline this is ‘all adult (19 plus years)’. Controlled vocabulary will be used for variant spellings, truncations and wildcards. Search strategies will be translated accordingly across the respective databases. Search terms were identified in consultation with our faculty librarian based on the PICOS framework. Table 1 displays the main search strategy tailored for Ovid Medline and is based on: primary outcome AND intervention type AND study design. Terms for PTSD (line 2) and terms to locate studies employing a RCT design (line 5‐8) are taken from a published Cochrane search strategy by Simon et al. (2021). To maximize the identification of studies not employing a RCT design, we utilize part of a search strategy published to identify non‐RCTs (Waffenschmidt et al., 2020; lines 10–11), as well as terms used by Simon et al. (2021) to identify waitlist and/or treatment as usual designs (see line 9).

**Table 1:** Example search strategy, adapted for Ovid MEDLINE.

1	Exp Stress Disorders, Post‐Traumatic/
2	(PTSD or ((posttrauma* or post‐trauma* or post trauma*) adj3 (stress* or disorder* or psych* or symptom? or Neuroses)) or acute stress disorder* or combat disorder* or war neuros*).ti,ab,kf.
3	Exp Psychotherapy, Group/
4	(group* adj5 (treat* or intervention* or therap* or train* or program* or session* or setting* or factor* or process* or counsel* or psychotherap* or psychoeducat* or psycho‐educat*)).mp.
5	Controlled clinical trial.pt.
6	Randomi#ed controlled trial.pt.
7	(RCT or at random or (random* adj3 (assign* or allocat* or control* or crossover or cross‐over or design* or divide* or division or number))).ti,ab,kf.
8	trial.ab,ti,kf.
9	(control* and (trial or study or group*) and (placebo or waitlist* or wait* list* or ((treatment or care) adj2 usual))).ti,ab,kf,hw.
10	exp cohort studies/or exp epidemiologic studies/or exp clinical trial/or exp evaluation studies as topic/or exp statistics as topic/
11	((control and (group* or study or trial)) or (time and factors) or program or survey* or ci or cohort or comparative stud* or evaluation studies or follow‐up*).mp.
12	or/1‐2
13	or/3‐4
14	or/5‐11
15	12 and 13 and 14
16	(animals/not humans/)
17	15 NOT 16
18	limit 17 to (english language and ‘all adult (19 plus years)’)

#### 3.2.1 Electronic searches

A number of databases have been identified and will be searched using the specified search terms in Table 1 adapted for each specific database, these include: (i) PsychINFO (via EBSCO), (ii) MEDLINE (via Ovid), (iii) Cochrane Central Register of Controlled Trials (CENTRAL), (iv) Embase (via the Elsevier platform), (v) PTSDpubs (via ProQuest, formally PILOTS), and (vi) Applied Social Sciences Index & Abstracts (ASSIA, via ProQuest). Search strategies for each database will be presented in Supporting Information. If unpublished studies are found within these databases they will also be eligible for inclusion. Grey literature will be searched for dissertations and unpublished articles, specifically searches will be conducted on Open Access Theses and Dissertations, ProQuest dissertations & theses, and preprint servers (PsyArXiv and medRxiv). Speciality journals will also be hand searched, including the European Journal of Psychotraumatology, the Journal of Traumatic Stress, and Psychological Trauma: Theory, Research, Practice, and Policy. We will search the Cochrane Database of Systematic Reviews (CDSR) to identify potential studies, ongoing trials, and/or unpublished trials. We will also search the PTSD Trials Standardized Data Repository (PTSD‐Repository; https://ptsd-va.data.socrata.com/) and a number of recommended websites (Kugley et al., 2017), including: the Government of Canada publications (https://publications.gc.ca/), Grey Literature Report (http://www.greylit.org/), and the National Institute for Health and Care Excellence (NICE; https://www.nice.org.uk/) for eligible studies.

#### 3.2.2 Searching other resources

Previous meta‐analyses and reviews of group‐based treatments for PTSD will be searched to identify any relevant studies (e.g., Barrera et al., 2013; Ehring et al., 2014; Schwartze et al., 2019; Sloan et al., 2013). We will also search the reference lists of any included studies to check for further relevant studies. To search for unpublished articles that may not be listed on the specified databases we will put out a call on Twitter asking for information on unpublished studies or unpublished data that fits the aims of the review and mentioning organizations that focus on PTSD in such tweets. Similarly, we will also contact international researchers by posting this call for information on the electronic mailing lists of a number of professional psychology societies and/or asking such societies to share this call with their members, such as the Society of Personality and Social Psychology, the American Psychosomatic Society, the Society of Affective Science, the European Association of Social Psychology, the Stress, Trauma, Anxiety, and Resilience Society (STAR), the International Society for Traumatic Stress Studies, and the European Society of Traumatic Stress Studies (and affiliated societies).

### 3.3 Data collection and analysis

#### 3.3.1 Description of methods used in primary research

We expect many studies will employ a RCT of some form that will consist of a group‐based treatment intervention condition and some other type of comparison condition (e.g., an individual‐based treatment or a control/waitlist/usual care condition). However, it may be the case that quasi‐experimental designs are employed due to the logistics of RCTs. For instance, participants may be allocated to a group‐based intervention or a waitlist based on recruitment timing (e.g., participants allocated to the intervention, then assigned to the waitlist once the intervention group is full). Studies need to assess PTSD symptoms pre‐ and post‐intervention.

#### 3.3.2 Criteria for determination of independent findings

A number of potential issues have been identified and planned for in this review. First, if multiple publications exist using the same data only the most complete report of these data will be included; if necessary, information will be collated across reports, but the study will be reported as a single unit of interest. Second, studies may include multiple outcome measures assessing the same construct or include multiple intervention arms (e.g., two group treatments and one control), this creates dependencies in the mean effects and this is planned for by the use of a correlated‐hierarchical effects (CHE‐RVE) model (Pustejovsky & Tipton, 2022) which accounts for such dependencies. However, in terms of multiple outcomes, clinician measures of PTSD take precedence over self‐reported PTSD measures, as previously outlined. For studies that include multiple intervention arms, we will only include the intervention arms/conditions that meet the eligibility criteria. Third, for studies that report outcome data at multiple follow‐up timepoints, we will model time differences using CHE‐RVE models, most likely by grouping time‐points as: immediately post‐intervention (or within 1 week after the last group session), short‐term follow‐up (1–12 weeks post‐intervention), medium‐term follow‐up (3–12 months post intervention), and long‐term follow‐up (>12 months post‐intervention).

#### 3.3.3 Selection of studies

After deduplication, each title and abstract will undergo preliminary screening independently by two authors. Studies that clearly do not match the eligibility criteria will be excluded. If a study is deemed eligible by one author (but not both), or if the study's eligibility is unclear from the title and abstract, then these studies will be rescreened in the full text screening phase. Likewise, each full text will be screened independently by two authors for eligibility. By completing the preliminary screening and full text screening independently this helps minimize bias. Both screening processes will be piloted by the review team. Studies outside of our criteria (e.g., do not assess PTSD symptoms pre‐ and post‐intervention, do not include a group‐based intervention) will be excluded. Any disagreements will be resolved through discussion and consensus, and if necessary, by asking a third author to screen the study independently. The screening process (e.g., number of hits from each database, number of duplicate records removed, number of records screened, records assessed for eligibility) and reasons for exclusion will be documented and presented in a PRISMA 2020 flow chart (Page et al., 2021). Covidence software will be used to support study screening.

#### 3.3.4 Data extraction and management

Two authors will independently extract the outcome data from each study to reduce the risk of error and potential bias. Both reviewers will follow a set of guidelines and complete a standardized data extraction sheet. This data extraction sheet will be developed and piloted with a random sample of 10 records to assess its validity, and will be revised as needed. In the case of any disagreements a consensus will be reached through discussion, and if not a third author will be consulted until consensus is reached. However, if a large number of studies are to be included, the lead author will extract the data and a random sample of the studies will be drawn and coded by a different team member to check the reliability of the coding to minimize the demandingness of the task.

Data extracted will include: (i) study metadata (authors, title, year, journal of publication), (ii) study characteristics (study design, sample size, study setting, measurement points, participants per group, participants per measurement point, average group size), (iii) participant information (age, gender, and any other relevant information), (iv) trauma characteristics (all participants experienced the same trauma or a mix of traumas, if the trauma was caused by intentional human action/s or not, if the trauma could be perceived as stigmatized or not), (v) intervention information (type of treatment used, assignment to intervention, number of participants per group, number of group sessions, duration of each group session, type of facilitator, number of facilitators, gender of participants in the group – mixed, all female, all male), (vi) comparison condition information (type of treatment or control, duration), (vii) any inclusion or exclusion criteria employed by the study authors (e.g., use of medication, comorbid conditions), (viii) primary and secondary outcomes (e.g., effect sizes, how measured), (ix) any group‐based factors (e.g., social support, group cohesion, trust), and (x) limitations of the study. Extracted data will be reported in the review in table format.

#### 3.3.5 Assessment of risk of bias

To support the assessment of the risk of bias of each study the Revised Cochrane risk‐of‐bias tool for randomized trials (RoB 2; Sterne et al., 2019) will be employed for RCTs and the Risk of Bias in Non‐randomized Studies—of Interventions (ROBINS‐I; Sterne et al., 2016) will be used for non‐RCTS. If any studies include a cluster‐randomized trial design, the Cochrane RoB 2 for cluster‐randomized trials will be used for these studies. These tools include a number of questions to assess the risk of bias within each study (e.g., randomization, incomplete data, selective outcome reporting).

Two authors will independently assess the risk of bias of each study and in the case of disagreement a third author will be consulted. Studies judged as having a critical risk of bias using the ROBINS‐I tool will be excluded from the meta‐analysis, consistent with recommendations (Sterne et al., 2016). As there is no critical level of risk within the RoB 2 tool, any RCTs judged as too problematic to provide useful evidence of the intervention will be excluded due to a critical risk of bias. As such, we will add a critical level of risk of bias to the RoB 2, similar to past reviews (Dalgaard et al., 2022). Consistent with this approach (Dalgaard et al., 2022), if a high risk of bias is determined in multiple domains with the RoB 2 or ROBINS‐I tool then this may lead to the study being deemed as at a critical risk of bias, and excluded from the meta‐analysis.

As we are interested in the group nature of the treatments, methodological heterogeneity will exist across studies in terms of the type of psychological treatment employed, the length of follow‐up, and the way in which groups were facilitated, therefore studies with a ‘high’ risk of bias will be included. However, we will conduct sensitivity analyses by excluding studies assessed as at a higher risk of bias to see if this affects the results. For the purposes of this review high risk of bias is indicated if the study is deemed ‘high risk’ using the RoB 2 tools or judged ‘serious’ using the ROBINS‐I tool (see Sensitivity Analyses plan). Risk of bias assessment will be reported in the Summary of Findings tables. Further, risk of bias will be discussed in the synthesis of study results and conclusions.

#### 3.3.6 Confounding

We have identified PTSD symptomology at baseline as a critical confounder. For RCTs we have planned for this in our analyses (e.g., preference to baseline adjusted effect sizes, using the pre–post‐test correlation when adjusted effect sizes are not reported). For non‐RCTs, these studies need to ensure baseline equivalence otherwise the study needs to provide results from which baseline‐adjusted effect sizes can be calculated; if not, the study will be excluded due to a critical risk of bias.

#### 3.3.7 Measures of treatment effect

Only studies that report continuous measures of PTSD are eligible for inclusion. Both within‐group effect sizes and between‐group effect sizes, and 95% confidence intervals, will be calculated. Between‐group effect sizes will be calculated for each comparison, each assessment time‐point, and each outcome of interest.

Effect estimates will be quantified as the standardized mean difference (SMD) by extracting the relevant data (e.g., means, sample sizes, standard deviations). Between‐group effect sizes will be computed using Hedges adjusted *g* using the small sample size bias correction (Hedges & Olkin, 1985), see Equation (1), where the difference between the mean outcome for the intervention and the comparison group is divided by the pooled within‐group standard deviation. Use of the standardized mean difference allows for comparisons to be made across groups when variables are not operationalized in the same way.

(1) $$\mathrm{SMD}=\frac{M_{G1}-M_{G2}}{s_{p}}\left(1-\frac{3}{4N-9}\right).$$

In studies were means or standard deviations are not reported, effect sizes will be calculated using other data within the article (e.g., standard errors, confidence intervals, *t* value, *p* value) using the methods and tools suggested by Lipsey and Wilson (2001) and Pustejovsky (2016).

We expect for objective 4, the proposed moderator analyses, that we may not have sufficient studies to conduct moderator analyses for between‐group effects. Therefore, we will calculate within‐group effect sizes for the intervention groups of each study, and examine moderators within pre‐post analyses, taking pre–post‐test correlations into account (WWC, 2021), as this approach will allow for increased power and remove the confounding effect of comparator conditions (similar to; Schwartze et al., 2019).

As all included studies will report pre‐ and post‐test outcomes on the same measure, if adjusted postintervention means are reported these will be used as a first preference, alongside unadjusted standard deviations (WWC, 2020). If only unadjusted means are reported, then the pre–post‐test correlation will be taken into account, by employing the difference‐in‐differences adjustment (WWC, 2021). In the case of RCTs, where the adjusted postintervention mean and/or the correlation is not available then the pre–post‐test correlation will be imputed (a value of 1.0 in estimating the effect size and a value of 0.5 in estimating its variance), in line with recommendations (WWC, 2021). For non‐RCTs, if baseline equivalence cannot be established, these studies will be excluded. If both adjusted effect sizes and unadjusted effect sizes are included in the meta‐analysis, then will we control for this in our analyses.

To be considered clinically relevant, the group‐based intervention will need to demonstrate an effect size of >0.80 for waitlist and usual care comparisons, >0.5 for attention control comparisons, >0.4 for placebo control comparisons, and >0.2 for active treatment comparisons, consistent with the definition of clinical importance (International Society for Traumatic Stress Studies [ISTSS], 2018; Lewis et al., 2020).

It may be the case that multiple effect sizes are extracted from each study (e.g., group intervention vs. control vs. individual intervention), therefore to account for dependency in the data we will use a Correlated and Hierarchical Effects Model (CHE‐RVE; Pustejovsky & Tipton, 2022), in line with the decision tree presented in Pustejovsky and Tipton (2022). This model combines the strengths of hierarchical effects models and correlated effects models to allow for between‐ and within‐study heterogeneity, as well as correlations between the effect size estimates in each study. For subgroup analyses, we will focus primarily on subgroup correlated effects (SCE) model (Pustejovsky & Tipton, 2022). Software for storing data and statistical analyses will be Review Manager 5 software (RevMan 5; The Cochrane Collaboration, 2022), Microsoft Excel, and R Statistical Software (R Core Team, 2021).

#### 3.3.8 Unit of analysis issues

Effect sizes will be computed for outcomes within each study. If a study provides more than one effect size for each outcome, this can be accounted for in statistical analyses. If there are more than two intervention arms, we will only include those that meet the review criteria. If we include a number of arms, we will ensure that these are independent samples (i.e., participants only took part in one arm) and report on only eligible arms and their effects.

Participants may have been randomized, or allocated, into the group‐based intervention or comparator groups in clusters. To minimize bias, we will apply a cluster bias correction (WWC, 2021), in addition to the small sample size adjustment. If no intra‐class correlation (ICC) is provided we will impute the ICC at 0.1 as recommended (WWC, 2020). In this scenario, we will conduct sensitivity analysis with changed ICC values. If the average group size is not obtainable, we will divide the total sample in the study by the number of groups included.

#### 3.3.9 Dealing with missing data

Study authors will be contacted if data of interest are not reported. If data are not available, we will not impute values. In the case of missing data due to follow‐up attrition (e.g., from pre‐ to post‐intervention) we will follow the principles of intention‐to‐treat analyses as much as possible. If the study authors use imputation in reporting their effect sizes these studies will be considered, however we will conduct sensitivity analyses to see if this influences the overall pattern of results. We will report on the extent of missing data narratively in the review and when discussing the risk of bias.

#### 3.3.10 Subgroup analysis and investigation of heterogeneity

We will examine if the following affect the observed results and potentially explain any observed heterogeneity: (i) trauma‐group type (members of the group experience the same trauma vs. a range of different traumas), (ii) stigma (the trauma experienced is stigmatized vs. not), (iii) the nature of the trauma (caused by the intentional actions of another person/s vs. not), (iv) gender grouping of the group‐based treatment (mixed gender grouping vs. all female vs. all male), (v) if the group was led by a clinician/facilitator or a peer, and (vi) group size (<6, 6‐8, >8). However, this is only possible if there are a sufficient number of studies for this to be meaningful (at least two studies per group). We will also conduct subgroup analyses with comparison condition as a factor (individual‐based treatment vs. no treatment) to examine the relative effect of these conditions. For categorical moderator variables, we will use the Subgroup‐Correlated Effects (SCE) models for subgroup analyses (Pustejovsky & Tipton, 2022). Based on past literature (Schwartze et al., 2019), there may not be enough studies to conduct subgroup analyses using between‐group effect estimates. In this case we will conduct subgroup analyses using the pre‐post effect sizes for the intervention groups.

We will assess the presence and extent of between‐study heterogeneity in a number of ways. In terms of methodological heterogeneity, this will be discussed narratively within the review as we anticipate a range of group‐based treatments to be used across studies. We anticipate some degree of heterogeneity due to methodological differences across group‐based intervention treatments. Therefore, for our analyses we will use CHE(‐RVE) models which takes into account both within‐study heterogeneity (ω) and between‐study heterogeneity (*τ*) and we will report on this data. Forest plots will be inspected to visualize the extent of heterogeneity (by examining the width of confidence intervals and degree of overlap). A forest plot aligned with dependent effect sizes will be used, similar to Winters et al. (2022). While we have already identified subgroup analyses which we believe will contribute to any possible heterogeneity, other subgroup analyses may be explored if needed (e.g., treatment type employed).

#### 3.3.11 Sensitivity analysis

Before conducting the analyses, we will check for outliers in the distribution of effect sizes. Outliers for the present review are effect size estimates that are more than three times outside the inter‐quartile range below the first quartile, or those more than three times the inter‐quartile range above the third quartile (Tukey, 1977; Winters et al., 2022). If outliers are present, we will conduct sensitivity analyses by removing these outliers and assess if this influences the results; if so, all analyses will be conducted with these outliers removed. Sensitivity analyses will be also be conducted to assess if the following influence results: (i) studies with a high risk of bias by rerunning the analysis excluding high risk studies (i.e., judged ‘high risk’ using the RoB 2 tool or ‘serious’ or using the ROBINS‐I tool; studies assessed as at a critical level of bias were already excluded from the meta‐analysis), (ii) the study design (i.e., inclusion of only RCTs vs. non‐RCTs), and (iii) studies with imputed data (where appropriate). If many group‐treatments include individual sessions (alongside a group intervention) we will conduct sensitivity analyses to see if this influenced the overall results.

#### 3.3.12 Treatment of qualitative research

This review will not include qualitative research as this is not the focus of the present review.

#### 3.3.13 Reporting bias

Egger's test for funnel plot asymmetry will be used to test for possible publication bias (Egger et al., 1997). Funnel plots will be presented visually and inspected.

## CONTRIBUTION OF AUTHORS

- Content: Siobhán M. Griffin, Orla T. Muldoon, Alžběta Lebedová, Grace McMahon, & Daragh Bradshaw.
- Systematic review methods: Siobhán M. Griffin, Alžběta Lebedová, Elayne Ahern & Daragh Bradshaw.
- Statistical analysis: Siobhán M. Griffin, Alžběta Lebedová, & Elayne Ahern.
- Information retrieval: Siobhán M. Griffin, Alžběta Lebedová, Grace McMahon, Daragh Bradshaw, Elayne Ahern, & Orla T. Muldoon.

## DECLARATIONS OF INTEREST

SG, OM, GM, and AL are involved in an ERC funded project that examines social group membership and trauma. This is an ongoing project from February 2021 to February 2026. This project does not interfere with the proposed review; rather the results of this review may be of value to the project by identifying what, if any, group‐based processes influence treatment outcomes for PTSD. EA and DB have no conflicts of interest to declare. We have a strong and experienced team and have no conflicts of interest related to studies that might be included in the present systematic review and meta‐analysis (conflict of interest forms for all authors will be collected at the protocol stage).

## PRELIMINARY TIMEFRAME

Anticipated submission date of the systematic review: September 2023.

## PLANS FOR UPDATING THE REVIEW

The search will be rerun every 5 years and the results screened for potentially eligible studies. This is the primary responsibility of the lead author, Siobhán M. Griffin, unless all authors agree at a later stage another author should take responsibility for updating the review.

## SOURCES OF SUPPORT

**External sources**. This research is supported by the European Research Council (ERC) under an Advanced Grant (Grant agreement No. 884927) awarded to Prof Orla Muldoon; SMG, AL, and GM are funded as part of this grant.
